# O.4.3-7 Mothers’ experiences of supporting their daughter to be physically active: a qualitative analysis

**DOI:** 10.1093/eurpub/ckad133.193

**Published:** 2023-09-11

**Authors:** Carol Brennan, Grainne O' Donoghue, Alison Keogh, Ryan E Rhodes, James Matthews

**Affiliations:** School of Public Health, Physiotherapy, and Sports Science, University College Dublin, Dublin, Ireland; School of Public Health, Physiotherapy, and Sports Science, University College Dublin, Dublin, Ireland; School of Medicine, Trinity College Dublin, The University of Dublin, Ireland; School of Exercise Science, Physical and Health Education, University of Victoria, Canada; School of Public Health, Physiotherapy, and Sports Science, University College Dublin, Dublin, Ireland; Institute of Sport and Health, University College Dublin, Dublin, Ireland; carol.brennan1@ucdconnect.ie

## Abstract

**Purpose:**

Girls are more at risk of non-communicable diseases associated with insufficient levels of physical activity (PA). Maternal support plays an important role in girl’s PA. Yet little is known about how mothers provide support to keep their daughters active. The aim of this study was to explore mothers’ experiences of supporting their daughter to be physically active.

**Methods:**

A qualitative study with semi-structured interviews was conducted with a purposive sample of mothers (n = 29) of girls (Mean age =10.9 years; SD = 0.6). Reflexive Thematic Analysis was used to analyse the data, with themes/subthemes mapped to the relevant domains of the Theoretical Domains Framework (TDF).

**Results:**

Most mothers recognised the importance of supporting their daughter to be active. Themes highlighted how a mother’s identity and confidence in supporting their daughter to be active influenced supportive behaviours. Social interactions played an important role in facilitating or inhibiting how a mother supports her daughter. The circumstances of a mother’s environment and her perceptions of her daughter’s attitudes towards PA influenced the type of support mothers provided.

**Conclusion:**

The findings advance our understanding of maternal PA support behaviours which will inform strategies to enhance maternal PA support with the ultimate goal of improving girls’ participation in PA. Future research should explore the appropriate behaviour change techniques for maternal PA support and involve mothers in the co-design process to develop interventions that are more feasible, acceptable and implementable.

